# Contemplative-based interventions for premenstrual syndrome: a systematic review of psychobiological outcomes

**DOI:** 10.3389/fpsyg.2026.1912206

**Published:** 2026-08-10

**Authors:** Sofía Walsen, Covadonga Chaves, Constanza Baquedano

**Affiliations:** 1 Center for Social and Cognitive Neuroscience (CSCN), School of Psychology, Adolfo Ibáñez University, Santiago, Chile; 2 Department of Personality, Assessment and Clinical Psychology, Complutense University of Madrid, Madrid, Spain

**Keywords:** contemplative practice, mindfulness meditation, mindfulness-based intervention, premenstrual syndrome, qigong, yoga

## Abstract

**Introduction:**

Premenstrual syndrome (PMS) is a cyclically recurring psychobiological condition affecting up to 47.8% of women of reproductive age, with considerable impact on daily functioning, quality of life and a documented economic burden linked to work absenteeism. Despite its prevalence, PMS remains substantially undertreated, and pharmacological approaches are often associated with undesirable side effects.

**Methods:**

This systematic review was conducted following PRISMA 2020 guidelines and evaluated the efficacy of contemplative practices (CPs), specifically mindfulness-based programs, yoga, and qigong, across psychobiological outcome domains in women with PMS. A comprehensive search of PubMed, Scopus, and Web of Science (2015–2026) identified 18 studies comprising 15 independent trials (*n* = 1,080).

**Results:**

Across studies, CPs were associated with improvements in mood, stress, and anxiety, reductions in somatic complaints, and enhanced sleep quality. Movement-based CPs (yoga and qigong) produced broader physiological effects including reductions in blood pressure, preliminary evidence of hormonal modulation, and cardiopulmonary improvements, while non-movement mindfulness-based interventions more selectively targeted psychological and emotional domains. Methodological quality was moderate (PEDro median = 7), and most trials were rated as having some concerns on RoB 2.0.

**Discussion:**

These findings are interpreted within a dual-axis modulation framework, in which CPs may simultaneously attenuate HPA-driven suppression of reproductive function and support HPO axis activity via autonomic and anti-inflammatory pathways. Future research should prioritize luteal phase-confirmed designs, objective biomarkers including heart rate variability and hormonal profiles, and adequately powered trials to elucidate mechanisms and optimal dosing.

**Systematic review registration:**

https://osf.io/hdpyf/overview.

## 1 Introduction

Premenstrual syndrome (PMS) affects approximately 47.8% of the worldwide population of women of reproductive age, presenting a range of emotional, cognitive, behavioral, and somatic symptoms that significantly interfere with women’s daily functioning, interpersonal relationships, and overall quality of life (Direkvand-Moghadam et al., 2014; Modzelewski et al., 2024; Maity et al., 2026; Liguori et al., 2023). These symptoms occur cyclically during the luteal phase and remit with the onset of menstruation, frequently disrupting work productivity, academic performance, and social participation (Gudipally and Sharma, 2022). The economic burden of this disruption is considerable: moderate to severe symptoms have been associated with a threefold increase in the likelihood of work productivity impairment and higher rates of absenteeism across multiple countries (Heinemann et al., 2010), and an average annual increase of nearly $4,500 in indirect costs per patient compared with women without the condition (Borenstein et al., 2005), a pattern recently corroborated in working women, where premenstrual disorders were associated with significantly worse work productivity and functional capacity (Loukzadeh et al., 2024). Beyond these direct economic consequences, women under the age of 45 show a cyclical 28-day pattern of workplace absenteeism due to menstruation that accounts for approximately 14% of the gender earnings gap (Ichino and Moretti, 2009), underscoring how PMS contributes to broader structural inequalities in women’s economic participation.

The American College of Obstetrics and Gynaecology (ACOG, 2001) has established criteria for moderate to severe PMS, requiring the presence of at least one psychological or physical symptom that leads to significant functional impairment, confirmed through prospective ratings (Yonkers et al., 2008). Moreover, the *Diagnostic and Statistical Manual of Mental Disorders* (DSM-5) recognizes premenstrual dysphoric disorder (PMDD) as a severe variant of PMS characterized primarily by mood-related symptoms. Despite differences between diagnostic systems, most women included in scientific studies on clinically significant PMS typically correspond to those meeting both ACOG and PMDD criteria (ACOG, 2001; American Psychiatric Association, 2013). However, some researchers question whether all symptoms occurring in the luteal phase should be conceptualized as a single syndrome, since, although there is general agreement that they are triggered by fluctuations in sex steroids and resolve when hormonal cyclicity ends, there is no definitive evidence that they share a single pathophysiological mechanism (Yonkers et al., 2008). Despite this clinical significance, PMS/PMDD remains substantially underrecognized and undertreated, with up to 89% of affected women going undiagnosed and its burden of disease estimated to be of comparable magnitude to that of major recognized conditions (Halbreich et al., 2003). This underrecognition reflects a broader tendency to normalize cyclically recurring symptoms that at their most severe are sufficiently disabling to impair occupational functioning and daily life representing a preventable and largely unaddressed burden in women’s healthcare.

**Figure 1:**
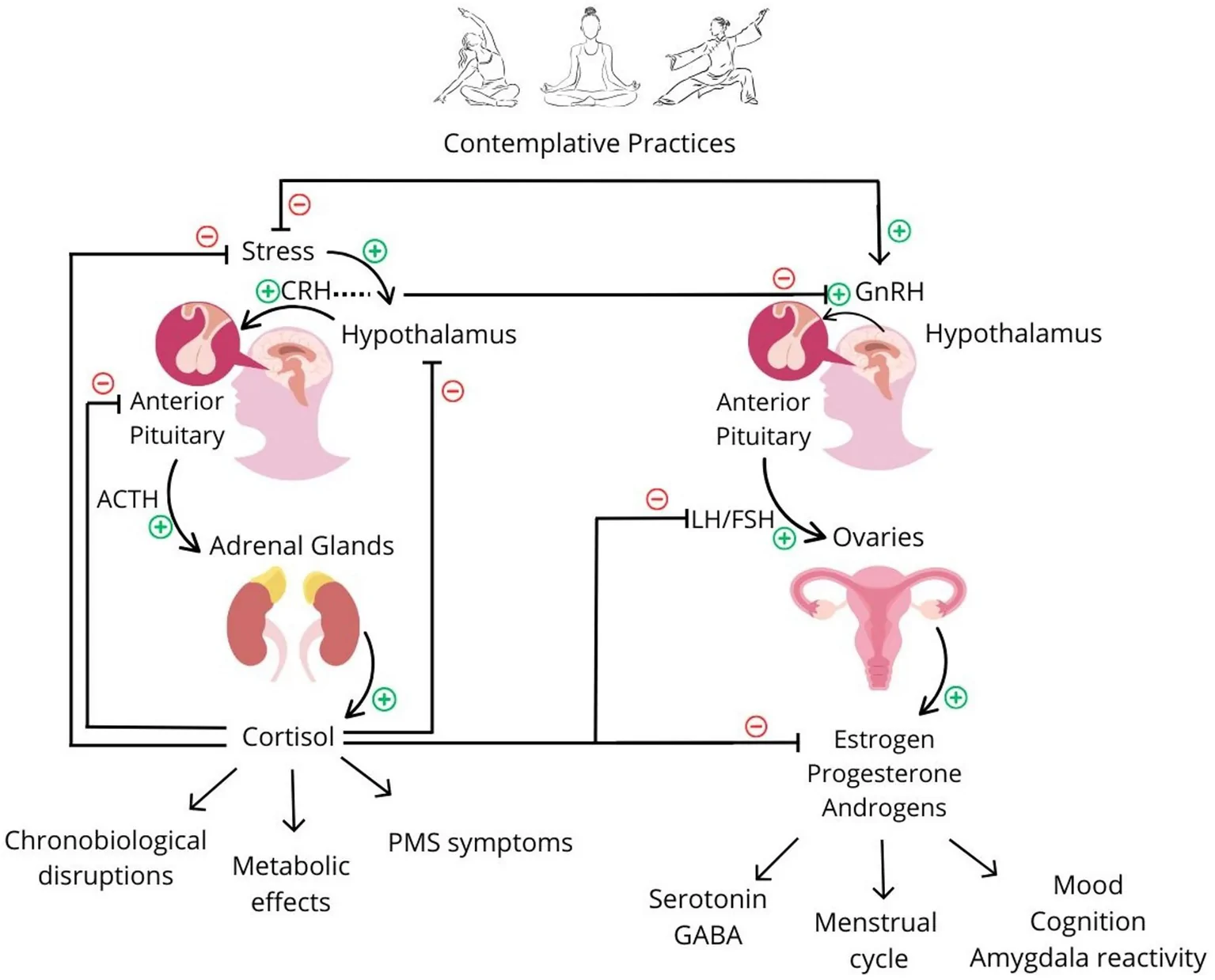
Dual-axis modulation framework illustrating the crosstalk between the hypothalamic–pituitary–adrenal (HPA) and hypothalamic–pituitary-ovarian (HPO) axes in PMS, and the proposed simultaneous inhibitory and facilitatory effects of contemplative practices on each axis. ⊕ activating; ⊖ inhibitory.

Although its etiology is multifactorial, PMS is understood to arise from an interaction between ovarian hormones and the brain’s stress and emotion regulation systems (Liu et al., 2024). Fluctuations in estrogen and progesterone across the menstrual cycle modulate neurotransmitter systems such as serotonin and GABA, with effects on emotionally relevant brain regions including the amygdala and prefrontal cortex; women with PMS show altered amygdala structure and connectivity during the luteal phase, with the strength of these changes correlating with symptom severity (Deng et al., 2018; van Wingen et al., 2008). This hormonal-neural sensitivity is embedded within a broader rhythm: the hypothalamic–pituitary-ovarian (HPO) axis drives the cyclical secretion of estrogen and progesterone that defines the follicular, ovulatory, and luteal phases (Plant, 2015), while interacting bidirectionally with the hypothalamic–pituitary–adrenal (HPA) stress axis (Tsigos et al., 2020; Figure 1). This HPA-HPO crosstalk is phase-dependent: during the luteal phase, women with PMS often show impaired regulation of the HPA axis, which may amplify stress sensitivity and contribute to the affective, somatic, and sleep-related symptoms characteristic of the condition, alongside signs of autonomic imbalance such as reduced heart rate variability (Hou et al., 2019; Blaser et al., 2024; Ozgocer et al., 2022). Together, this dual-axis crosstalk provides the proposed psychobiological framework within which the relevance of interventions targeting both stress regulation and hormonal synchrony can be understood (Figure 1).

Pharmacological treatments such as selective serotonin reuptake inhibitors, benzodiazepines, and hormonal contraceptives have shown efficacy in reducing PMS symptoms, but are frequently associated with undesirable effects, including dependency risk, sexual dysfunction, and, in the case of hormonal contraceptives, an increased risk of depression among adolescents and first-time users (Ghaffarilaleh et al., 2019a; Dimmock et al., 2000; Skovlund et al., 2016). Given these limitations, there is increasing interest in non-pharmacological alternatives such as contemplative practices (CPs).

Although no single consensus definition has been established in the literature (Dorjee, 2016), CPs are broadly understood as forms of training that emphasize self-awareness, self-regulation, and/or self-inquiry to enact a process of psychological transformation (Davidson and Dahl, 2017). In cognitive and affective neuroscience, the term has gained traction as a unifying category for the diverse traditions increasingly studied in empirical research (Josipovic and Baars, 2015), encompassing practices such as mindfulness meditation, yoga, and qigong. These converge on a common orientation toward cultivating well-being and emotional balance through shared attentional and self-regulatory mechanisms (Lutz et al., 2008), while differing in their emphasis on movement, breathwork, and the degree of somatic versus cognitive engagement. For the purposes of this review, CPs are operationally defined as structured and intentional practices incorporating at least one of the following components: sustained attentional training, breath regulation, body-based awareness, or meditative inquiry, a definition that encompasses mindfulness-based interventions (MBIs), yoga, and qigong as its principal instantiations.

Preliminary evidence suggests that these three modalities act on a shared psychobiological terrain implicated in PMS. Yoga has been associated with improvements in mood, blood pressure, and sleep in women with PMS (Misra and Chetri, 2024), mindfulness-based programs have shown reductions in anxiety and emotional reactivity in menstrually related mood disorders (Bluth et al., 2015; Şener Çetin and Şolt Kırca, 2023), and qigong-based practices such as Baduanjin have been linked to reductions in psychological and physical symptom severity in independent cohorts (Zhang et al., 2015; Vaghela et al., 2025). From a psychobiological perspective, these effects are thought to arise from the simultaneous engagement of neural, endocrine, and autonomic systems, including attenuation of HPA axis hyperactivation, support of HPO function, strengthened vagal tone, and improved prefrontal-limbic regulation (Leung et al., 2017; Blaser et al., 2024). This convergence across modalities and systems is what makes contemplative practice psychobiologically plausible as a unifying intervention for PMS, offering a possible counterpoint to the dual-axis dysregulation described above.

The effects that CPs have on PMS may be partially explained by mechanisms such as direct stress reduction, enhanced cognitive functioning, interoceptive awareness, increased functional connectivity within emotion regulation networks, and improved HRV (Bluth et al., 2015; Borlimi et al., 2023). Such effects are coherent with the theoretical framework of “embodied synchronization” (Rodríguez-Muguruza, 2023), which proposes that cyclical bodily rhythms can be harmonized through regular practices of bodily awareness that are socially shared. In this sense, contemplative engagement can be understood as one such pathway, fostering coherence across psychobiological domains.

This evidence, however, remains conceptually fragmented, with no unified psychobiological framework available to interpret how HPA, HPO, and autonomic systems may interact with one another in the context of contemplative practice for PMS. This fragmentation is compounded by a lack of systematic synthesis. Although some randomized controlled trials (RCTs) have explored CPs for PMS management, their findings have not been systematically synthesized across psychophysiological domains, and prior systematic reviews have examined yoga in isolation (Pal et al., 2022; Oates, 2017), without integrating the full spectrum of outcomes and CPs within a unified framework.

This review situates the existing findings within a biopsychosocial model of menstrual health, aiming to synthesize empirical evidence on the efficacy of mindfulness-based and CPs for PMS, with particular attention to psychological, behavioral and physiological outcomes. Therefore, this paper examines which psychobiological processes underlying PMS symptoms can be modulated by CPs. Framed in PICOS terms, this review addressed the following question: among women of reproductive age with PMS (Population), do mindfulness-based, yoga or qigong (Intervention), compared with no intervention, usual care, or active comparators (Comparator), improve psychological, physiological or behavioral outcomes (Outcomes), as assessed in randomized controlled trials or non-randomized controlled studies (Study design)?

To address this question, we conducted a systematic review to evaluate the effects of CPs, particularly mindfulness-based, yoga and qigong, for the management of PMS, and to analyze their impact across psychobiological dimensions. Accordingly, the following specific objectives were formulated (1) To analyze the characteristics of the interventions (e.g., type, duration), (2) To examine the characteristics of the participants (e.g., age, diagnosis, menstrual phase), (3) To explore the effects of the interventions from a psychobiological perspective (e.g., psychological, physiological and behavioral level), (4) To analyze potential moderators of intervention effects (if such information was reported).

## 2 Methods

This systematic review adhered to the PRISMA 2020 guidelines (Page et al., 2021). A comprehensive search was conducted in May 2026 across three electronic databases: PubMed, Scopus and Web of Science to evaluate the efficacy of CPs, particularly mindfulness-based and yoga, for the management of PMS and to analyze their impact across psychobiological dimensions.

### 2.1 Search terms

employed in the literature review included combinations of: (“contemplative practice” OR “mindfulness-based intervention” OR “mindfulness meditation” OR “yoga” OR “taichi” OR “qigong”) AND (“premenstrual syndrome” OR “PMS”). Boolean logic and filters for publications between 2015 and 2026 were applied (See Supplementary Table 1 for specific search strings, filters and retrieved record counts).

### 2.2 Eligibility criteria

Studies were considered suitable if they fulfilled the following criteria: (1) Randomized controlled trials or non-randomized controlled studies (e.g., allocation by diagnostic/clinical criterion) evaluating CPs for PMS; (2) Interventions incorporating CPs such as mindfulness, yoga, or qigong; (3) Published in peer-reviewed journals in English between 2015 and 2026; (4) Participants are women of reproductive age with specialized instruments or clinically diagnosed PMS; (5) Intervention duration between 4 and 24 weeks; (6) Reported outcomes across psychobiological domains (psychological, physiological and/or behavioral).

### 2.3 Study selection

**Figure 2:**
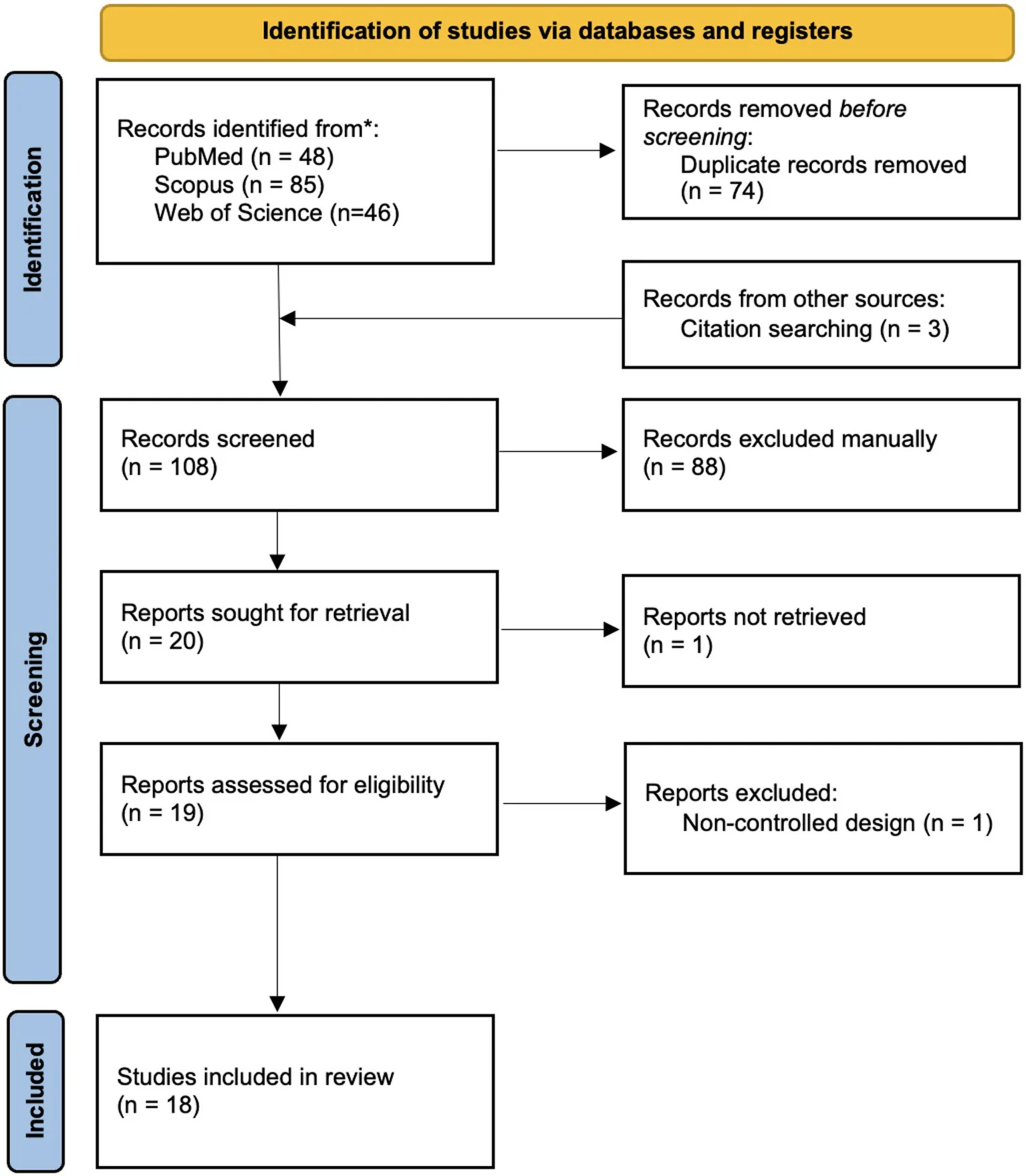
Study selection process illustrated by a PRISMA flow diagram. A total of 179 records were initially identified from three databases: PubMed (*n* = 48), Scopus (*n* = 85) and Web of Science (*n* = 46). After removing 74 duplicates manually and including 3 records from citation searching, 108 records were screened. Of these, 88 were excluded manually based on title and abstract relevance. A total of 19 full-text articles were assessed for eligibility, with one subsequently excluded. The final review included 18 studies. *Reflects that record counts are reported separately by database (PubMed, Scopus, Web of Science).

A total of 179 records were identified, 48 on PubMed, 85 on Scopus and 46 on Web of Science. Seventy-four duplicates were removed and three records from citation searching were included. Titles and abstracts of 108 studies were screened for eligibility independently by two reviewers (S. W. and C. B.), with disagreements resolved by consensus. Exclusion during this stage was due to non-controlled design (*n* = 1). Following a detailed assessment, only 18 studies fulfilled the inclusion criteria for the final synthesis (see Figure 2 for details on the study selection).

### 2.4 Data extraction

Data were extracted including citation information (author(s), date published, country of study), sample information (number of participants, mean age, participant characteristics), intervention type (mindfulness, yoga or qigong), intervention characteristics (duration, adherence, and satisfaction metrics), outcome domains assessed (psychological or biological). Where possible, psychobiological outcomes were grouped into three categories: psychological (self-report), behavioral and physiological.

Due to the substantial heterogeneity across included trials in intervention type, outcome measures, assessment instruments, and follow-up timing, a meta-analytic synthesis was not considered appropriate. This heterogeneity was evident across several dimensions; Intervention type varied considerably across type of CPs, protocol delivered, duration, and intensity. Outcome measures also varied widely, drawing on a range of psychological, physiological and behavioral instruments with limited overlap across studies. For instance, while several trials relied on the Premenstrual Syndrome Scale or the Moos Menstrual Distress Questionnaire, physiological outcomes ranged from heart rate variability parameters to hormonal panels and electroencephalographic measures, each reported in only a few studies. Follow-up timing differed as well, with assessment windows ranging from immediately post-intervention to several menstrual cycles later. Results are therefore presented as a narrative synthesis following SWiM (Synthesis Without Meta-analysis) guidelines, organized by intervention category and outcome domain, with effect sizes reported where available to support qualitative interpretation of the magnitude of observed differences.

### 2.5 Quality assessment

Methodological quality of RCTs was assessed using the 11-item Physiotherapy Evidence Database (PEDro) scale to index internal validity and statistical interpretability (Maher et al., 2003). Items cover eligibility criteria, random allocation, concealed allocation, baseline comparability, blinding of participants, therapists, and outcome assessors, ≥85% follow-up for at least one key outcome, intention to treat (or complete case with no dropouts), between-group statistical comparisons, and point/variability estimates. For self-administered outcomes, assessor blinding was considered met when participants were blinded. Items not clearly reported were scored 0. All PEDro ratings were assigned independently by two reviewers (SW and CB), with disagreements resolved through discussion and consensus. Where agreement could not be reached, a third reviewer was consulted.

Additionally, risk of bias was assessed using the revised Cochrane Risk of Bias tool for randomized trials (RoB 2.0; Sterne et al., 2019), applied to each study’s primary outcome. This tool evaluates five domains: (1) bias arising from the randomization process, (2) bias due to deviations from intended interventions, (3) bias due to missing outcome data, (4) bias in measurement of the outcome, and (5) bias in selection of the reported result. Each domain was rated as low risk, some concerns, or high risk. Ratings were assigned independently by two authors, with discrepancies resolved through iterative discussion and consensus. In cases of persistent disagreement, a third reviewer was consulted to reach a final determination. Given the participatory and non-concealable nature of CPs, participant and provider blinding was structurally impossible across all included trials; accordingly, Domain 4 judgments reflect the susceptibility of self-reported outcomes to performance and detection bias rather than inadequate study conduct. The aim of the assessment was to estimate the effect of assignment to intervention (intention-to-treat effect).

## 3 Results

### 3.1 Study characteristics

A total of 18 articles reporting on 15 independent trials, comprising 1,080 participants, were included in the final synthesis (Table 1). Across trials, CPs duration ranged from 4 to 24 weeks and included mindfulness-based programs (e.g., MBSR; 8 sessions and a 6 h silent retreat), mindfulness counseling (8 weekly sessions, 60 min), an app-based mindfulness program (daily practice over 8 weeks), yoga (typically 10 to 12 weeks, 3 sessions a week, 60 min each), yoga and acupressure (12 sessions once a week for 60 min), Hatha yoga (12 sessions over 6 weeks), and Baduanjin qigong (daily, 30 min sessions over 24 weeks, comprising an initial 12-week supervised phase followed by 12 weeks of self-directed practice). Mindfulness programs emphasized sustained attention, body awareness (interoception), and a non-judgmental stance; yoga protocols integrated breath regulation (prāṇāyāma), postures (āsana), and relaxation/meditation; Baduanjin employed eight slow, breath-coordinated movements with focused attention. Notably, the papers by Kamalifard et al. (2017) and Ghaffarilaleh et al. (2019a, 2019b, 2019c) were identified as multiple reports deriving from a single clinical trial (IRCT201501216582N9).

**Table 1:** Characteristics of studies.

Author and References	Intervention	Duration	Participants	Variables	Main results
Şener Çetin and Şolt Kırca (2023)	Mindfulness-Based Stress Reduction (MBSR)	8 w, 8 sessions + 6 h retreat	*n* = 90 (45 E, 45 C)	PMSS	↓ PMSS total (*p* < 0.001)
Hojjati Najafabadi et al. (2023)	Mindfulness counseling	8 w, 8 sessions (60 min)	*n* = 112 (56 E, 56 C)	FSFI, PSST	↑ sexual function in the intervention group (*p* < 0.0001)
Mazaheri Asadi et al. (2022)	Mindfulness-Based Intervention (MBI) Mobile App	8 w, every day	*n* = 80 (40 E, 40 C)	GHQ-28, PSST, SF-12	↓ PSST total (*p* < 0.001), ↓ PSST mood and behavioral/physical symptoms scores in intervention group (*p* < 0.001). ↑ SF-12 physical and mental health in intervention group (*p* < 0.001)
Alkhatib et al. (2024)	Qigong Baduanjin	24 w, every day (30 min)	*n* = 62 (31 E, 31 C)	MSQ, SCAS, PSS, PSQI, Blood hormone tests	↓ MSQ total score (*p* < 0.01), ↓ scores for the two subscales of MSQ: premenstrual symptoms (*p* < 0.01) and menstrual pain (*p* < 0.05). ↓ SCAD, perceived stress level, and ↑ sleep quality. ↑ Progesterone and estrogen (*p* < 0.05)
Abic et al. (2024)	Yoga and Progressive Muscle Relaxation (PMR)	8 w	*n* = 68 (17 Yoga, 17 PMR, 17 Yoga + PMR, 17 C)	PMSS, DASS-21	↓PMS scores in Yoga (*p* = 0.039) and Yoga+PMR (*p* < 0.001) groups. ↓ DASS-21 in all active groups (*p* ≤ 0.047); Yoga+PMR superior to control for depression (*p* < 0.001) and anxiety (*p* = 0.019), and to PMR for stress (*p* = 0.044) and anxiety (*p* = 0.012)
Ghaffarilaleh et al. (2019a)	Yoga	10 w, 3 sessions a week (60 min)	*n* = 62 (31 E, 31 C)	PSQI	↑ Sleep quality PSQI (*p* = 0.001)
Ghaffarilaleh et al. (2019b)	Yoga	10 w, 3 sessions a week (60 min)	*n* = 62 (31 E, 31 C)	PSST, BDI-II, Diastolic pressure	↓ BDI-II (p = 0.036), ↓ diastolic blood pressure (*p* = 0.036)
Ghaffarilaleh et al. (2019c)	Yoga	10 w, 3 sessions a week (60 min)	*n* = 62 (31 E, 31 C)	HARS, Diastolic pressure	↓ anxiety total score (*p* < 0.05) and specific HARS subdomains: anxiety, tension, fears, insomnia, depression, somatic, cardiovascular, respiratory, intellectual, and autonomic symptoms (*p* < 0.05)
Feula et al. (2024)	Pranayama (yoga breathwork)	8 w, 5 days/week (20 min)	*n* = 40 (10 E PMS, 10 C PMS, 20 no-PMS)	CAFT, HRV (SDNN, RMSSD, LFnu, HFnu, LF/HF), BRS, P300, MOCA	↓ mean HR, SBP, LFnu (*p* < 0.001); ↑ HFnu, SDNN, RMSSD, BRS (*p* = 0.011); ↓ P300 latency (*p* = 0.002); ↑ MOCA (*p* < 0.001)
Joshi and Bhilare (2025)	Yoga	8w	*n* = 30 (15 E, 15 C)	NPRS, MAF, 6MWT	↓ Pelvic pain (NPRS) and ↓ fatigue (MAF) post intervention in the experimental group compared to control (*p* = 0.0001)
Kamalifard et al. (2017)	Yoga	10 w, 3 sessions a week (60 min)	*n* = 62 (31 E, 31 C)	PSST	↓ PSST all emotional, physical and behavioral variables between yoga and control groups (*p* < 0.001). ↓ impact of PMS on the life after yoga (*p* < 0.001)
Lata and Lohan (2018)	Yoga	10 w, 6 days a week (45 min)	*n* = 60 (30 E, 30 C)	MMDQ	↓ all MMDQ scales (pain, water retention, autonomic reactions, etc.) and total PMS score in yoga group compared to control (*p* < 0.05)
Li et al. (2026)	Yoga	12 w, 2 sessions a week (60 min)	*n* = 64 (32 E, 32 C)	Dot-probe task, EEG 64-ch NeuroScan (P1, P3), PSST, PARS	↓ attention orientation toward negative stimuli (*p* < 0.001). ↓ attention disengagement difficulty (*p* < 0.001). ↓ P1 amplitude (*p* < 0.001). ↓ P3 amplitude (*p* = 0.002). Attention orientation negatively correlated with P1 (*p* < 0.001); attention disengagement positively correlated with P3 (*p* = 0.046).
Ranga and Dev (2026)	Yoga and Pilates	6 w, 3 sessions a week (45 min)	*n* = 36 (12 Yoga, 12 Pilates, 12 C)	PSST, Blood pressure (SBP, DBP), Heart rate, Respiratory parameters, QoL	Yoga produced greatest improvements. ↓ SBP (*F* = 7.592, *p* = 0.002), DBP (*p* < 0.001), HR (*p* < 0.001). ↑ FVC, FEV1, FEV1/FVC, PEFR (*p* < 0.001). ↓ depression (*p* = 0.003), anxiety (*p* < 0.001), stress (*p* = 0.003). ↑ psychological QoL (*p* < 0.001); physical, social, environmental domains non-significant.
Simsek Kucukkelepce et al. (2020)	Yoga and Acupressure (Acu)	12 w, Yoga: 1 session a w (60 min); Acu: 2 sessions a w (60 min)	*n* = 155 (50 Yoga, 51 Acu, 54 C)	PMSS, WHOQOL-B	↓ PMSS scores (*p* < 0.05). Significant differences in the physical health, psychological health, and environment sub-scales of the WHOQOL-B (*p* < 0.05).
Wu et al. (2015)	Hatha Yoga	6w, 12 sessions	*n* = 20 (11 E, 9 C)	EEG (portable 40-channel), Cognitive task (2-back task), PAF, MMDQ	↑ alpha wave percentage of the PMS group showed a significant effect through yoga exercise (*p* = 0.01), higher after yoga. ↑ performance accuracy after yoga in the PMS group (*p* = 0.01) and in reaction time in follicular phase in PMS group (*p* < 0.01) and after yoga (*p* = 0.02)
Yildiz Karaahmet et al. (2026)	Yoga	8 w, 3 sessions a week (40 min)	*n* = 131 (65 E, 66 C)	PMSS, VAS, Blood parameters (E2, progesterone, prolactin, CRP, NEU, LYM, BASO, haematocrit, sedimentation)	↓ PMSS total scores (*p* < 0.001, η^2^ = 0.967). ↓ all nine PMSS subdomains (*p* < 0.05). ↓ VAS pain scores (*p* < 0.001, η^2^ = 0.842). ↓ E2, progesterone, and prolactin (all *p* < 0.001). ↓ CRP, NEU, LYM, BASO, sedimentation, and haematocrit (all *p* < 0.05). No significant changes in haemoglobin, red blood cell count, or eosinophils.
Yorulmaz et al. (2024)	Long-term regular Yoga	12w, 3 sessions a week (45 min)	*n* = 60 (30 E, 30 C)	PMSS, Visual Analog Scale (VAS) for pain, WHOQOL-B	↓ PMSS total (*p* = 0.010), ↓ Pain severity (VAS; *p* = 0.002), ↑ Quality of life (WHOQOL-B) in the yoga group compared to control (*p* ≤ 0.026).

E, Experimental group; C, Control group; w, Number of weeks of intervention; PMSS, Premenstrual Syndrome Scale; FSFI, Rosen Female Sexual Functioning Index; PSST, Premenstrual Symptoms Screening Tool; GHQ-28, General Health Questionnaire; SF-12, 12-Item Short-Form Health Survey; WHOQOL-B, Short form of the World Health Organization Quality of Life Questionnaire; BDI-II, Beck Depression Inventory-second edition; HARS, Hamilton Anxiety Rating Scale; PAF, Premenstrual Assessment Form; PARS, Physical Activity Rating Scale; MMDQ, Moos Menstrual Distress Questionnaire; MSQ, Menstrual Symptom Questionnaire; SCAS, Sociocultural Adaptation Scale; PSS, Perceived Stress Scale; PSQI, Pittsburgh Sleep Quality Index; DASS-21, Depression Anxiety Stress Scale-21; NPRS, Numerical Pain Rating Scale; MAF, Multidimensional Assessment of Fatigue; 6MWT, Six-Minute Walk Test; EEG, Electroencephalography; SBP, Systolic Blood Pressure; DBP, Diastolic Blood Pressure; HR, Heart Rate; FVC, Forced Vital Capacity; FEV1, Forced Expiratory Volume in 1 s; PEFR, Peak Expiratory Flow Rate; CAFT, Cardiovascular Autonomic Function Tests; HRV, Heart Rate Variability; SDNN, Standard Deviation of Normal-to-Normal intervals; RMSSD, Root Mean Square of Successive Differences; LFnu/HFnu, Normalized low/high frequency HRV power; LF/HF, Low-to-high frequency HRV ratio; BRS, Baroreflex Sensitivity; MOCA, Montreal Cognitive Assessment; VAS, Visual Analog Scale; E2, Estradiol; CRP, C-Reactive Protein; NEU, Neutrophil count; LYM, Lymphocyte count; BASO, Basophil count; ↓, Significant decrease; ↑, Significant increase.

### 3.2 Participant characteristics

The studies recruited women with PMS, predominantly university students to early adulthood (Table 1), with diagnosis based on validated tools such as the Premenstrual Syndrome Screening Tool (PSST), Premenstrual Syndrome Scale (PMSS), and the Moos Menstrual Distress Questionnaire (MMDQ). In some trials, phase-aware assessment was performed (e.g., luteal phase measurements). Sample sizes ranged from small acute studies (*n* = 20) to large trials (*n* = 155), conducted across Turkey, Iran, Taiwan, India, and China.

### 3.3 Methodological quality

Across the included trials, PEDro total scores ranged from 5 to 7 (median 7, mean 6.4), indicating moderate methodological quality. Eligibility criteria (item 1) were universally reported, and all trials provided between-group statistical comparisons (item 10) and point or variability estimates (item 11). Random allocation (item 2) was reported in most trials, while allocation concealment (item 3) was documented in only two trials, Kamalifard et al. (2017) and Ghaffarilaleh et al. (2019a, 2019b, 2019c), which used sealed opaque envelopes prepared by a non-involved researcher, and Simsek Kucukkelepce et al. (2020) who employed an equivalent procedure. Blinding of participants (item 5) and therapists (item 6) was absent across all trials, consistent with the participatory nature of CPs. Intention-to-treat analysis was explicitly reported in Kamalifard et al. (2017), Joshi and Bhilare (2025), and Ranga and Dev (2026); notably, Mazaheri Asadi et al. (2022) explicitly acknowledged a per-protocol design. Follow-up rates below the 85% threshold were observed in Mazaheri Asadi et al. (2022; 67%). Full item-level ratings are presented in Supplementary Table 2.

Risk of bias assessed with RoB 2.0 revealed a nuanced picture that warrants careful interpretation considering the inherent characteristics of CPs research. Domain 4 (measurement of the outcome) was rated as “some concerns” across the majority of included trials, reflecting the structural impossibility of blinding participants and providers to participatory interventions. This is a constraint shared by all behavioral and exercise-based research rather than a reflection of inadequate study conduct. Given this field-wide limitation, studies were rated “high risk” overall only when additional methodological concerns were present in other domains, independent of the unblindable nature of the intervention. Under this approach, three studies were rated as high risk overall. Mazaheri Asadi et al. (2022) received high risk ratings in Domains 2 and 3 due to the per-protocol analysis design combined with 33% attrition in both arms, and in Domain 4 given the absence of any objective outcome measure. Şener Çetin and Şolt Kırca (2023) were rated high risk in Domain 1 due to the absence of allocation concealment and in Domain 4, as self-reported outcomes under unblinded conditions with no objective corroboration heightened susceptibility to performance bias. Wu et al. (2015) were rated high risk in Domain 1, as group assignment was determined by diagnostic criteria rather than randomization, and received some concerns in Domain 4 given the inclusion of objective neurophysiological outcomes alongside subjective report.

The remaining 15 studies were rated “some concerns” overall, primarily driven by the unavoidable Domain 4 exposure to performance and detection bias, but without additional domain-level concerns that would justify a high-risk classification. Domain 1 (randomization) was rated as low risk in trials that described adequate random sequence generation and allocation concealment, including Kamalifard et al. (2017), Ghaffarilaleh et al. (2019a, 2019b, 2019c), Simsek Kucukkelepce et al. (2020), Ranga and Dev (2026), Joshi and Bhilare (2025), and Yorulmaz et al. (2024). Domain 5 (selection of reported results) was rated low risk in four prospectively registered trials (Şener Çetin and Şolt Kırca, 2023, NCT05191108; Joshi and Bhilare, 2025, CTRI/2022/09/045735; Ranga and Dev, 2026, CTRI/2023/09/057303; and Mazaheri Asadi et al., 2022, IRCT20180607040000N2) and as some concerns in the remaining studies due to the absence of pre-specified analysis plans. Notably, Li et al. (2026), Wu et al. (2015), and Feula et al. (2024) employed objective neurophysiological outcomes (EEG event-related potentials and reaction times), which partially mitigates the detection bias inherent in self-report designs and distinguishes them from the broader pattern of outcome measurement in this literature. Full domain-level ratings are presented in Supplementary Table 3.

### 3.4 Synthesis of results

#### 3.4.1 Mindfulness

##### 3.4.1.1 Self-report outcomes

Three RCTs examined the effects of mindfulness-based interventions on PMS-related outcomes using exclusively self-report measures. An 8-week MBSR program delivered online significantly reduced overall PMS symptoms compared to controls with significant between-group differences across seven of nine symptom subscales, including anxiety, fatigue, irritability, and depressive feelings (Şener Çetin and Şolt Kırca, 2023). Similarly, an 8-week smartphone-based mindfulness program similarly reduced overall PMS symptoms (PSST) and improved physical and mental health-related quality of life (SF-12) across mood and behavioral/physical symptom subscales, though high attrition (33%) and a per-protocol design limit the strength of these conclusions (Mazaheri Asadi et al., 2022).

Extending the scope of mindfulness outcomes beyond symptom burden, eight sessions of online mindfulness counseling significantly improved multiple domains of sexual functioning in women with PMS compared to controls, including desire, orgasm, satisfaction, sexual pain, and overall sexual functioning, with gains maintained at one-month follow-up (Hojjati Najafabadi et al., 2023). Sexual arousal reached significance only at follow-up, while vaginal lubrication did not differ significantly between groups at either timepoint. These findings suggest that mindfulness may support sexual functioning in PMS.

#### 3.4.2 Qigong

##### 3.4.2.1 Self-report outcomes

A single RCT evaluated Baduanjin qigong in international female students undergoing acculturation stress (Alkhatib et al., 2024). Over 24 weeks of daily 30-min practice, the intervention group showed significant reductions in overall menstrual symptom burden compared to controls (MSQ), with specific improvements in premenstrual symptoms and menstrual pain. Beyond menstrual symptomatology, perceived stress (PSS), sociocultural adaptation, and sleep quality (PSQI) also improved significantly, with sleep gains emerging progressively across both the supervised and self-directed phases.

##### 3.4.2.2 Physiological outcomes

Blood hormone concentrations assessed at week 24 showed significant increases in progesterone and estrogen in the Baduanjin group relative to controls, while control values remained stable (Alkhatib et al., 2024). These hormonal changes co-occurred with reductions in menstrual symptom severity. Adherence was high (≥85% session completion) with no adverse events recorded.

#### 3.4.3 Yoga

##### 3.4.3.1 Self-report outcomes

Four publications derived from a single RCT conducted in Iran (IRCT201501216582N9) reported outcomes across distinct symptom domains; findings should be interpreted jointly rather than as independent replications (Kamalifard et al., 2017; Ghaffarilaleh et al., 2019a, 2019b, 2019c). Ten weeks of Hatha yoga produced significant between-group reductions across all emotional, physical, and behavioral PMS symptom domains, alongside improvements in depression (BDI-II), anxiety (HARS), and sleep quality (PSQI). Anxiety improvements spanned 11 of 14 HARS subdomains, and sleep gains were specific to subjective quality, latency, and efficiency, with duration and daytime dysfunction remaining unchanged.

Convergent evidence for broad symptom reduction comes from two further trials with distinct active comparators. A 10-week yoga program produced significant reductions across all eight MMDQ domains compared to controls, including pain, negative affect, impaired concentration, and total score (Lata and Lohan, 2018). However, the absence of allocation concealment and a within-group analytic emphasis limits the strength of these conclusions. By contrast, a three-arm RCT comparing yoga, acupressure, and a no-intervention control found yoga superior to both comparator groups in overall PMS symptom reduction, with significant improvements across physical, psychological, and environmental WHOQOL-BREF domains but not social relationships (Simsek Kucukkelepce et al., 2020). Acupressure showed significant change only in the physical subscale.

Two trials with active control conditions further characterized the physical and psychological reach of yoga. A supervised RCT focused on pelvic pain and fatigue found no significant between-group differences at 4 weeks, but significant reductions in both outcomes at 8 weeks (Joshi and Bhilare, 2025). A pilot RCT comparing yoga, Pilates, and pelvic floor exercise similarly found yoga superior to both comparators for depression, anxiety, and stress, with only psychological quality of life showing a significant between-group difference among the WHOQOL domains; retention was 100% and adherence 97.22% with no adverse events (Ranga and Dev, 2026).

A single-blinded RCT comparing long-term regular yoga practitioners against non-practitioners found significantly lower PMSS total scores in the yoga group, with significant between-group differences across six of nine subscales: depressive feelings, anxiety, fatigue, depressive thoughts, pain, and sleep habits (Yorulmaz et al., 2024). Pain severity was significantly lower in the yoga group, and quality of life was higher across all four WHOQOL-BREF domains. However, the cross-sectional comparison design limits direct inference about change from baseline, as acknowledged by the authors.

A four-arm parallel RCT compared yoga, PMR, their combination (yoga+PMR), and a no-intervention control in university students with PMS (Abic et al., 2024). PMSS total scores decreased significantly within all three active groups, while the control group showed no significant change. Between-group comparisons indicated that yoga and yoga+PMR had significantly lower PMSS scores than controls, whereas PMR alone did not differ significantly from controls. On the DASS-21, all three active conditions showed significant within-group reductions in depression, anxiety, and stress. Between-group analyses favoured yoga+PMR over controls for depression and anxiety, and over PMR alone for stress and anxiety, though the student sample and absence of follow-up limit generalizability.

In a single-blind RCT, the yoga group reported a significant and progressive reduction in total PMSS severity across all nine subdomains, encompassing depressive affect, anxiety, fatigue, irritability, depressive thoughts, menstrual pain, appetite changes, sleep disturbances, and bloating, while the control group showed no significant change over time (Yildiz Karaahmet et al., 2026). Pain intensity assessed by weekly VAS similarly decreased significantly in the yoga group throughout the intervention period, with no comparable change observed in controls.

##### 3.4.3.2 Physiological outcomes

Two reports from the same Tabriz trial assessed physiological parameters. Both found a significant reduction in diastolic blood pressure in the yoga group following the intervention, while systolic blood pressure, heart rate, and BMI did not change significantly in either group (Ghaffarilaleh et al., 2019b, 2019c).

The most comprehensive physiological assessment among yoga trials in this review compared yoga, Pilates, and pelvic floor exercise control over 6 weeks (Ranga and Dev, 2026). Within-group analyses showed significant reductions in systolic blood pressure, diastolic blood pressure, and heart rate in the yoga group, with between-group superiority confirmed over Pilates and control for all cardiovascular parameters. Pulmonary function also improved significantly within the yoga group across FVC, FEV1, FEV1/FVC ratio, and PEFR, with between-group differences favouring yoga for all respiratory parameters. The Pilates group showed significant but consistently smaller improvements in most cardiopulmonary variables relative to controls in this small pilot sample.

Extending the physiological scope of yoga trials in this review, a single blinded RCT simultaneously assessed hormonal, haematological, and inflammatory blood parameters alongside symptom outcomes (Yildiz Karaahmet et al., 2026). Estradiol, progesterone, and prolactin levels all decreased significantly following the intervention, with significant between-group differences favouring yoga, while no hormonal changes were observed in the control group. Inflammatory and haematological parameters also improved significantly in the yoga group, with reductions in CRP, neutrophil count, lymphocyte count, basophil count, erythrocyte sedimentation rate, and haematocrit, whereas haemoglobin, red blood cell count, and eosinophil levels remained unchanged.

##### 3.4.3.3 Behavioral outcomes

The acute neurophysiological and cognitive effects of a single yoga session were examined using EEG and a 2-back working memory task in women with PMS (Wu et al., 2015). It should be noted that group assignment in this study was based on diagnostic criteria rather than randomization, which limits causal inference. Immediately following yoga practice, resting alpha wave percentage increased significantly in the PMS group. On the 2-back task, yoga was associated with significant improvements in both accuracy and reaction time in the PMS group, with faster and more accurate responses observed post-exercise, particularly during the luteal phase when baseline performance was most impaired. Event-related potential analyses revealed a significant main effect of menstrual cycle phase on P3 amplitude in the PMS group, with higher amplitudes during the luteal phase, while no such cyclic variation was observed in controls.

Extending this line of inquiry, the effects of 12 weeks of regular yoga on attentional bias during the luteal phase were investigated using a dot-probe paradigm combined with EEG in 64 women with PMS randomly assigned to yoga or control conditions (Li et al., 2026). Repeated-measures ANOVA revealed significant time and group interaction effects for attention orientation, attention disengagement, P1 amplitude, and P3 amplitude. Simple effect analyses indicated that, compared to controls, the yoga group showed significant reductions in attentional orientation toward negative facial stimuli, significant decreases in P1 amplitude, improvements in attention disengagement, and reductions in P3 amplitude after 12 weeks. Cluster-based permutation tests corroborated these findings. Pearson correlation analyses further indicated that attention orientation was significantly negatively correlated with P1 amplitude, while attention disengagement was positively correlated with P3 amplitude. These findings suggest that regular yoga practice may modulate both the initial orienting response to threat-relevant stimuli and the capacity to disengage from them, with P1 reflecting early attentional capture and P3 indexing higher-order evaluative processing.

Complementing these electrophysiological findings, a pranayama-focused RCT provided converging evidence that breathwork-based CPs can concurrently modulate autonomic function and cognitive performance in women with PMS (Feula et al., 2024). The protocol combined assessments including HRV, BRS, P300 event-related potentials, and MOCA (Montreal Cognitive Assessment). At baseline, women with PMS showed elevated sympathetic indices and reduced parasympathetic markers relative to controls, alongside prolonged P300 latency and lower MOCA scores. Following the intervention, the pranayama group showed a significant shift toward parasympathetic dominance including reductions in LFnu, increases in HFnu, SDNN, RMSSD, and marked improvement in baroreflex sensitivity, alongside significant shortening of P300 latency and improvement in MOCA scores. No changes were observed in the no-intervention PMS group.

### 3.5 Adherence and satisfaction

Adherence and retention across the included trials were generally high. Completion rates exceeded 90% in most yoga trials, with attrition primarily attributable to pregnancy or personal circumstances rather than dissatisfaction (Ghaffarilaleh et al., 2019a, 2019b, 2019c; Kamalifard et al., 2017). The highest adherence was reported in Ranga and Dev (2026); 97.22% and Alkhatib et al. (2024); ≥85% session completion, with no adverse events in either trial. Feula et al. (2024) similarly reported complete data for all participants and no adverse events. The main exception was Mazaheri Asadi et al. (2022), where 33% attrition was attributed primarily to technical barriers with the app platform rather than to the intervention itself. Although explicit satisfaction measures were rarely reported, high retention rates across trials are consistent with good acceptability. Joshi and Bhilare (2025) and Ranga and Dev (2026) noted no adverse events, and participants in the latter trial expressed satisfaction with both yoga and Pilates. Together, these patterns are consistent with CPs across formats (app-based, group, supervised, and home-based) being generally well-tolerated, with high engagement reported across most trials.

## 4 Discussion

This systematic review presents preliminary evidence consistent with the potential efficacy of CPs, particularly mindfulness-based interventions, yoga and qigong, in improving emotional, behavioral, cognitive, and physiological symptoms associated with PMS. Across the included randomized controlled trials, participants consistently reported improvements in emotional regulation, stress, anxiety and somatic complaints, with several studies also documenting benefits in sleep quality, interpersonal functioning, and cognitive performance (Şener Çetin and Şolt Kırca, 2023; Ghaffarilaleh et al., 2019a; Mazaheri Asadi et al., 2022). These findings highlight the potential of CPs as non-pharmacological strategies for menstrual health, supporting psychophysiological wellbeing in women with PMS (Misra and Chetri, 2024).

### 4.1 Psychobiological mechanisms

The effects observed are consistent with the proposed psychobiological mechanisms underlying PMS and previous research in the area, which emphasize the role of HPA–HPO axis crosstalk in symptom generation. Hormonal fluctuations across the menstrual cycle and stress influence the autonomic nervous system and emotional regulation pathways, often leading to heightened sympathetic activity and reduced parasympathetic tone (Blaser et al., 2024). Neuroimaging studies further support this perspective, showing increased amygdala volume and altered connectivity with the medial prefrontal cortex and anterior cingulate cortex in women with PMS (Deng et al., 2018), as well as progesterone-induced modulation of amygdala reactivity (van Wingen et al., 2008). CPs may counterbalance this dysregulation by reducing perceived stress, enhancing vagal activity, downregulating the HPA axis, and supporting homeostatic functions (Abic et al., 2024; Erdoğan et al., 2024; Vargas-Uricoechea et al., 2024). Further research is needed to directly examine these proposed physiological pathways using objective markers such as HRV, cortisol, hormonal and neuroimaging measures.

**Figure 3:**
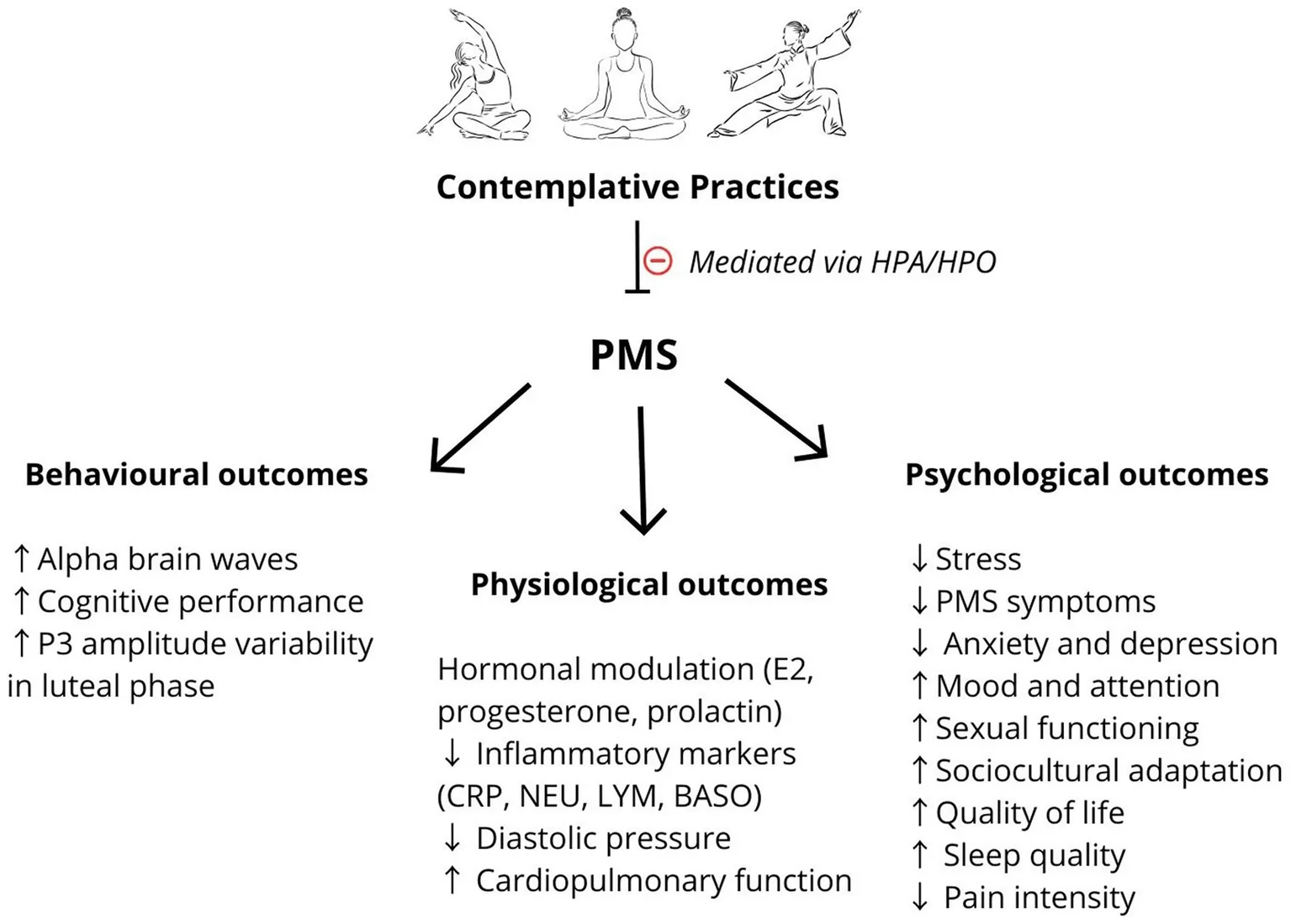
Psychobiological outcomes associated with CPs across the included trials, organized by domain (psychological, physiological, and behavioral).

These relationships are summarized in Figure 3, which integrates the present results into a conceptual framework where CPs may influence PMS through cognitive, biological, and psychological pathways. Hormonal findings across trials are best understood in light of the menstrual cycle phase at which measurements were obtained. Increases in progesterone and estrogen associated with Baduanjin qigong practice are consistent with enhanced HPO axis support during the luteal phase, when progesterone dominance is clinically desirable, with baseline progesterone values suggesting luteal-phase sampling, though cycle phase was not formally verified (Alkhatib et al., 2024). By contrast, reductions in estradiol, progesterone, and prolactin following yoga were assessed during the early follicular phase, and could plausibly reflect phase-appropriate hormonal normalization rather than suppression: reduced estradiol outside the luteal window may suggest improved metabolic clearance, while reduced follicular progesterone could tentatively indicate resolution of the HPO dysfunction that can produce inappropriately elevated levels in women with PMS (Yildiz Karaahmet et al., 2026). The reduction in prolactin may further point toward a possible attenuation of stress-driven neuroendocrine dysregulation via HPA pathways. Together, these findings are consistent with a dual-axis modulation hypothesis in which CPs may support restoration of normal hormonal cyclicity rather than uniformly suppressing or elevating individual hormones. Future research should prioritize measurement across both follicular and luteal phases within the same trial and examine the progesterone-to-estradiol ratio during the luteal phase as a clinically meaningful index of HPO axis synchrony. Psychological benefits indicate reduced stress, anxiety, and depressive symptoms, parallel neuroimaging findings of decreased right amygdala volume in experienced meditators and yoga practitioners (Leung et al., 2017; Gotink et al., 2018).

### 4.2 Cognitive and neurophysiological pathways

Cognitive and neurophysiological outcomes across three trials converge on an attentional regulation pathway through which CPs may modulate both cortical arousal and the processing of emotionally salient stimuli during the luteal phase. Acute yoga practice increased resting alpha wave activity and improved working memory accuracy specifically in women with PMS (Wu et al., 2015), while 12 weeks of regular yoga modulated electrophysiological correlates of attentional bias, with P1 reflecting automatic early capture of negative stimuli and P3 indexing higher-order evaluative processing (Li et al., 2026). Importantly, Feula et al. (2024) extended this evidence to breathwork-based practice, showing that pranayama concurrently improved P300 latency and MOCA scores alongside autonomic indices, suggesting that the cognitive benefits of CPs are not exclusive to movement-based yoga but may be shared across modalities that emphasize breath regulation and parasympathetic activation. Together, these findings may support the hypothesis that sustained contemplative engagement progressively influences attentional control networks in ways that reduce the neurocognitive load imposed by the luteal-phase emotional environment, with P3 amplitude modulation proposed as a potential shared electrophysiological marker of this process across modalities (Atchley et al., 2016).

### 4.3 Movement-based versus non-movement practices

Movement-based practices produced broader physiological and cognitive effects including reductions in blood pressure, cardiopulmonary improvements, hormonal modulation, and attentional bias changes, while non-movement mindfulness-based interventions acted more selectively on mood, anxiety, and sexual functioning (Ghaffarilaleh et al., 2019b; Ranga and Dev, 2026; Li et al., 2026; Alkhatib et al., 2024; Şener Çetin and Şolt Kırca, 2023). This differential profile appears broadly consistent with a plausible mechanistic distinction, as rhythmic movement may engage vagal afferent pathways and could potentially influence reproductive endocrine function via peripheral pathways, whereas attention-focused practices may act more selectively on prefrontal and insular networks implicated in interoceptive regulation and affective labeling (Alkhatib et al., 2024; Guendelman et al., 2017). Notably, pranayama produced both autonomic and cognitive improvements through breath regulation alone, suggesting that the physiological reach of CPs may depend less on gross motor engagement than on the degree to which the intervention activates parasympathetic pathways (Feula et al., 2024). These findings collectively suggest that movement and non-movement practices are complementary rather than interchangeable, and that their combination may yield additive benefits across physiological and psychological domains.

### 4.4 Temporal gradient of benefit

The evidence base also points toward a dose-dependent and temporally structured pattern of benefit. Acute effects of a single yoga session, increased resting alpha wave activity and improved working memory performance in the luteal phase, were reported by Wu et al. (2015) in a small sample, tentatively suggesting that even brief exposure could engage cortical regulation mechanisms relevant to cognitive function during the premenstrual window. At 6 to 8 weeks, consistent reductions in self-reported mood, anxiety, and somatic complaints emerged across yoga and mindfulness trials (Abic et al., 2024; Şener Çetin and Şolt Kırca, 2023; Ghaffarilaleh et al., 2019a–c), with Joshi and Bhilare (2025) reporting that clinically meaningful differences in pelvic pain and fatigue required at least 8 weeks to consolidate. Extended protocols of 10 to 24 weeks produced the broadest outcomes, encompassing physiological markers such as blood pressure and hormonal modulation (Alkhatib et al., 2024; Yorulmaz et al., 2024), neurophysiological changes in attentional processing (Li et al., 2026), and multidimensional quality of life improvements. This temporal gradient suggests that future trials should tailor their duration to the outcome domain of interest; acute or short-term protocols may suffice for cognitive and mood outcomes, while physiological and hormonal benefits may require more sustained engagement. Minimum effective doses and maintenance thresholds remain empirically undetermined and represent a priority for future research.

### 4.5 Beyond symptom relief

Beyond the reduction of discrete symptoms, several findings in this review point to interoceptive awareness as a candidate common pathway across contemplative modalities. The interventions that engaged the body as an explicit site of regulation (yoga, qigong, and mindfulness) were also those reporting gains in domains that extend past symptom counts: improvements in sexual functioning following mindfulness counseling (Hojjati Najafabadi et al., 2023), and concurrent gains in sociocultural adaptation, perceived stress, and sleep quality with Baduanjin qigong (Alkhatib et al., 2024). We interpret these patterns as preliminary and hypothesis-generating rather than established, the convergence is suggestive, but no included trial directly measured interoceptive accuracy, and the relational outcomes derive from single studies. With that caveat, the data are at least consistent with the proposal that contemplative training fosters emotional integration and buffers the interpersonal stressors that intensify during the luteal phase, in line with broader contemplative accounts emphasizing non-reactivity and relational presence (Bluth et al., 2015; Borlimi et al., 2023; Shabani and Khalatbari, 2019).

We further propose, as a conceptual frame for future testing rather than a conclusion supported by the present evidence, that these effects can be read through the lens of PMS as a state of “infradian desynchronization,” in which misalignment between biological rhythms, emotional regulation, and social context exacerbates symptom expression (Rodríguez-Muguruza, 2023). Within this frame, CPs may function as resynchronizing tools, operating not only on the psychobiological systems already discussed (HPA–HPO crosstalk and autonomic balance) but also on the experiential coherence between bodily state and awareness. Importantly, this resynchronization hypothesis remains untested: no trial in this review verified luteal-phase status biochemically, so whether CPs realign a desynchronized luteal state or simply attenuate chronic dysregulation cannot yet be distinguished. We therefore advance it as a generative model to be examined with the phase-confirmed, multi-axis designs outlined below.

### 4.6 Methodological quality

These substantive findings must, however, be interpreted considering the methodological characteristics of the evidence base. Methodological quality across the 18 included studies was moderate overall, with a pattern of strengths and limitations that constrains causal inference without undermining the consistency of the observed effects. PEDro scores (median 7; range 5–7) indicated adequate randomization and outcome reporting across most trials with persistent weaknesses in allocation concealment, participant and therapist blinding, and intention-to-treat analysis, all structural features of CPs research rather than study-specific failures.

The RoB 2.0 assessment yielded a more differentiated picture than a straightforward high-risk count would suggest. Domain 4 (measurement of the outcome) was rated as some concerns in the majority of studies, reflecting the structural impossibility of blinding participants and providers to participatory interventions, a field-wide constraint shared across exercise, psychotherapy, and behavioral research broadly rather than a reflection of inadequate study conduct. Consistent with this, high-risk overall ratings were reserved for studies where methodological concerns in additional domains compounded the measurement susceptibility. Three studies were rated high risk overall: one due to 33% attrition analyzed per protocol without intention-to-treat correction (Mazaheri Asadi et al., 2022), one due to the absence of allocation concealment alongside fully self-reported outcomes (Şener Çetin and Şolt Kırca, 2023), and one due to non-randomized group assignment by diagnostic criteria (Wu et al., 2015). The remaining 15 studies were rated some concerns overall, a classification that more accurately reflects the nature of the evidence than a blanket high-risk label. Studies employing objective neurophysiological or physiological outcomes alongside self-report represent the clearest methodological advance available within this field-wide constraint, pointing toward the value of pairing validated instruments with objective biological or electrophysiological markers to reduce dependence on subjective reporting and strengthen causal inference (Wu et al., 2015; Li et al., 2026; Feula et al., 2024).

### 4.7 Limitations and future directions

Despite promising results, several methodological limitations constrain the conclusions that can be drawn from this body of evidence. Many studies relied on self-reported diagnosis of PMS without prospective charting across multiple cycles, as recommended by ISPMD consensus criteria, which raises the possibility that some participants did not meet strict diagnostic thresholds. Critically, hormonal confirmation of menstrual cycle phase was absent in all but one study: only Alkhatib et al. (2024) measured sex hormone concentrations, and no trial verified luteal phase status biochemically at the time of outcome assessment. This is a significant gap given that PMS symptoms are, by definition, luteal-phase phenomena, and self-reported cycle phase estimates are subject to considerable variability. Without objective phase confirmation, it remains unclear whether the observed improvements reflect genuine modulation of the luteal-phase psychobiological state or a more general reduction in chronic dysregulation occurring with the menstrual cycle.

Beyond hormonal measurement, physiological outcomes were assessed in only a minority of trials, and markers with relevance to the proposed HPA–HPO crosstalk mechanisms, such as heart rate variability, cortisol profiles, and inflammatory markers, were not reported as primary outcomes in any included study. The simultaneous assessment of subjective symptom burden alongside these objective physiological indices would allow a more precise quantification of how CPs modulate each axis independently and in interaction, as the crosstalk between the HPA and HPO axes suggests that changes in one system are likely to reverberate through the other in ways that self-report instruments alone cannot disentangle. This mechanistic gap is compounded by the near-universal absence of hormonal data, which also limits understanding of a clinically relevant downstream consequence, the impact of chronic stress-related HPO dysregulation on reproductive function and fertility. Given that PMS reflects a phase-dependent vulnerability in the hormonal regulation of the cycle, interventions that modulate HPA axis hyperactivation may plausibly exert protective effects on reproductive health more broadly, yet this hypothesis remains entirely untested in the CPs literature. Sample sizes were often small, and few trials included long-term follow-up assessments, making it impossible to determine whether observed benefits persist beyond the intervention period or require continuous practice to be maintained.

A further limitation concerns the geographic and cultural concentration of the evidence base, with included trials conducted predominantly in Turkey, Iran, Taiwan, India and China, settings where CPs such as yoga and qigong are often more culturally embedded than many Western contexts. This concentration is unlikely to affect the physiological outcomes reported in this review, as mechanisms such as autonomic regulation and hormonal modulation are not expected to vary systematically with cultural context. However, culturally mediated factors, including symptom reporting norms, willingness to engage in CPs, and perceived acceptability of these interventions, may differ across settings and could influence psychological outcomes in ways not captured by the present evidence base. This gap highlights the need for research conducted in Western and other underrepresented cultural contexts, both to test whether the physiological and behavioral benefits observed generalize across populations and to examine how cultural factors may shape the different outcomes of CPs for PMS.

Considering these limitations, future research should prioritize objective hormonal verification of menstrual cycle phase to ensure outcomes genuinely reflect luteal-phase modulation. The simultaneous collection of physiological indices alongside validated self-report instruments would enable formal mediation analyses of HPA–HPO crosstalk, clarifying whether improvements in symptoms are mediated by autonomic shifts, hormonal normalization, or both. Extending this line of inquiry to fertility-relevant outcomes would open a clinically meaningful and currently neglected area of women’s health research. Beyond research design, integrating structured educational components such as menstrual cycle tracking and fertility awareness-based methods (Simmons and Jennings, 2020) could complement CPs and provide women with actionable tools to support long-term menstrual health. Moderator analyses are needed to identify which women benefit most from which type of practice and mixed-methods frameworks drawing on neurophenomenological approaches could clarify how subjective symptom relief maps onto neural and endocrine changes in ways that quantitative designs alone cannot capture.

To synthesize, the following methodological priorities are proposed for the field: (1) Standardized hormonal confirmation of menstrual cycle phase through progesterone assays, LH surge detection, or basal body temperature charting, to anchor outcome measurement to verified luteal-phase windows; (2) Integration of a minimum physiological dataset, including heart rate variability, salivary cortisol, and sex hormone concentrations, to enable quantification of HPA–HPO crosstalk and move beyond exclusive reliance on self-report; (3) Examination of fertility-relevant outcomes, including cycle regularity and ovulatory function, to explore whether stress-modulating CPs may have broader reproductive health implications; (4) Mixed-methods and neurophenomenological designs that pair neurophysiological measures such as ERP or neuroimaging with structured first-person accounts, to bridge the gap between objective neural change and the lived experience of practice; (5) Adequately powered trials with active control conditions, prospective registration, long-term follow-up, and moderator analyses to establish effective dosing parameters and identify which women benefit most.

## 5 Conclusion

Taken together, the evidence reviewed supports the potential of CPs as integrative, psychobiologically grounded and low-risk interventions for premenstrual syndrome. Across 18 articles reporting on 15 independent trials, yoga, mindfulness-based interventions and qigong were consistently associated with improvements in mood, anxiety, sleep quality and somatic complaints, with movement-based practices additionally showing potential effects on blood pressure, cardiopulmonary function and hormonal modulation.

Emerging electrophysiological evidence further suggests that sustained yoga practice may be associated with changes in attentional control networks during the luteal phase, potentially reflecting modulation of both early automatic capture of negative stimuli and the capacity to disengage from them, a finding with possible clinical relevance given the heightened emotional reactivity characteristic of PMS. These multidimensional benefits are consistent with a dual-axis modulation framework in which CPs may simultaneously be linked to reduced HPA-driven inhibition of reproductive function and support HPO axis activity through autonomic and inflammatory pathways, although the current evidence does not allow these relationships to be established with certainty. Movement-based and non-movement practices appear to be complementary rather than interchangeable and combining them may yield additive benefits across physiological and psychological domains. In sum, CPs emerge as promising, safe, and potentially useful strategies for the management of PMS, although evidence remains preliminary and requires confirmation through higher-quality studies with larger sample sizes and more comprehensive assessment of physiological and neuroendocrine variables.

As this field matures, prioritizing luteal-phase-confirmed designs, objective biomarkers and neurophenomenological frameworks will be essential to clarify mechanisms, establish effective dosing parameters and fully realize the therapeutic potential of these approaches for women’s menstrual health.

## Data Availability

The original contributions presented in the study are included in the article/Supplementary material, further inquiries can be directed to the corresponding author.
