# Supplementary material for: Contemplative-based interventions for premenstrual syndrome: a systematic review of psychobiological outcomes
Source: Front Psychol. 2026 Aug 10;17:1912206. doi: 10.3389/fpsyg.2026.1912206 (PMC13500289; doi:10.3389/fpsyg.2026.1912206)
Supplement: Supplementary file 1 [file Table_1.docx]

Supplementary Table 1. Search Strings

| **Database** | **Search String** | **Filters Applied** | **Records Retrieved** |
| --- | --- | --- | --- |
| PubMed | ((contemplative practice) OR (mindfulness-based intervention) OR (mindfulness meditation) OR (yoga) OR (taichi) OR (qigong)) AND ((premenstrual syndrome) OR (PMS)) | Publication Years 2015-2026 | 48 |
| Scopus | (TITLE-ABS-KEY(contemplative practice) OR TITLE-ABS-KEY(mindfulness based intervention) OR TITLE-ABS-KEY(mindfulness meditation) OR TITLE-ABS-KEY(yoga) OR TITLE-ABS-KEY(taichi) OR TITLE-ABS-KEY(qigong)) AND (TITLE-ABS-KEY(premenstrual syndrome) OR TITLE-ABS-KEY(pms)) AND PUBYEAR > 2014 | Publication Years 2015–2026 | 85 |
| Web of Science | TS=((contemplative practice OR "mindfulness based intervention" OR "mindfulness meditation" OR yoga OR taichi OR qigong) AND (premenstrual syndrome OR PMS)) Filters: Publication Years 2015–2026 | Publication Years 2015–2026 | 46 |
