# Supplementary material for: Contemplative-based interventions for premenstrual syndrome: a systematic review of psychobiological outcomes
Source: Front Psychol. 2026 Aug 10;17:1912206. doi: 10.3389/fpsyg.2026.1912206 (PMC13500289; doi:10.3389/fpsyg.2026.1912206)
Supplement: Supplementary file 2 [file Table_2.docx]

Supplementary Table 2. Summary of quality assessment of studies (PEDro Scale)

| Study | 1 | 2 | 3 | 4 | 5 | 6 | 7 | 8 | 9 | 10 | 11 | Total |
| --- | --- | --- | --- | --- | --- | --- | --- | --- | --- | --- | --- | --- |
| Abic et al. (2024) | 1 | 1 | 0 | 1 | 0 | 0 | 0 | 1 | 1 | 1 | 1 | 7 |
| Alkhatib et al. (2024) | 1 | 1 | 0 | 1 | 0 | 0 | 0 | 1 | 1 | 1 | 1 | 7 |
| Hojjati Najafabadi et al. (2023) | 1 | 1 | 0 | 1 | 0 | 0 | 0 | 1 | 0 | 1 | 1 | 6 |
| Joshi & Bhilare (2025) | 1 | 1 | 0 | 1 | 0 | 0 | 0 | 1 | 1 | 1 | 1 | 7 |
| Kamalifard et al. (2017) | 1 | 1 | 1 | 1 | 0 | 0 | 0 | 1 | 1 | 1 | 1 | 8 |
| Feula et al. (2024) | 1 | 1 | 0 | 1 | 0 | 0 | 0 | 1 | 0 | 1 | 1 | 6 |
| Ghaffarilaleh et al. (2019a) | 1 | 1 | 1 | 1 | 0 | 0 | 0 | 1 | 1 | 1 | 1 | 8 |
| Ghaffarilaleh et al. (2019b) | 1 | 1 | 1 | 1 | 0 | 0 | 0 | 1 | 1 | 1 | 1 | 8 |
| Ghaffarilaleh et al. (2019c) | 1 | 1 | 1 | 1 | 0 | 0 | 0 | 1 | 1 | 1 | 1 | 8 |
| Lata & Lohan (2018) | 1 | 1 | 0 | 0 | 0 | 0 | 0 | 1 | 1 | 1 | 1 | 6 |
| Li et al. (2026) | 1 | 1 | 1 | 1 | 0 | 0 | 1 | 1 | 0 | 1 | 1 | 8 |
| Mazaheri Asadi, Tajrishi & Gharaei (2022) | 1 | 1 | 0 | 1 | 0 | 0 | 0 | 1 | 0 | 1 | 1 | 6 |
| Ranga & Dev (2026) | 1 | 1 | 0 | 1 | 0 | 0 | 0 | 1 | 1 | 1 | 1 | 7 |
| Simsek Kucukkelepce et al. (2020) | 1 | 1 | 0 | 1 | 0 | 0 | 0 | 1 | 0 | 1 | 1 | 6 |
| Şener Çetin & Şolt Kırca (2023) | 1 | 1 | 0 | 1 | 0 | 0 | 0 | 1 | 0 | 1 | 1 | 6 |
| Wu et al. (2015) | 1 | 0 | 0 | 1 | 0 | 0 | 0 | 1 | 0 | 1 | 1 | 5 |
| Yildiz Karaahmet et al. (2026) | 1 | 1 | 0 | 1 | 0 | 0 | 1 | 1 | 0 | 1 | 1 | 7 |
| Yorulmaz et al. (2024) | 1 | 1 | 0 | 1 | 0 | 0 | 0 | 1 | 1 | 1 | 1 | 7 |

*Note.* Criteria: 1) Specified eligibility criteria; 2) Random allocation of subjects to groups; 3) Concealed allocation; 4) Groups similar at baselines on key prognostic indicators; 5) Blinding of all subjects; 6) blinding of all therapists administering treatment; 7) Blinding of all assessors measuring at least one key outcome; 8) Measurements of at least 85% of subjects; 9) Intention to treat analysis; 10) Results of statistical comparisons between groups; 11) Point and variability measures are provided in the study.
